# A 3D finite element model to simulate progressive damage in unidirectional- and woven-fibre thermoplastic discontinuous-long-fibre composites

**DOI:** 10.1177/08927057231158535

**Published:** 2023-03-01

**Authors:** Réjean Belliveau, Benoit Landry, Gabriel LaPlante

**Affiliations:** Département de génie mécanique, Université de Moncton, Moncton, NB, Canada

**Keywords:** Discontinuous-long-fibre composites, woven fibres, unidirectional fibres, finite element modelling, progressive damage

## Abstract

Discontinuous-long-fibre (DLF) composites fabricated from pre-impregnated unidirectional (UD) fibre chips are susceptible to structural deficiency. The in-plane highly anisotropic mechanical properties of the chips combined with the random nature of fibre orientation causes local weaknesses within the material when fibres are perpendicular to the load. Recent experimental results have shown that using woven-fibre chips could improve the performance of DLF composites by increasing their average mechanical properties and reducing their variability. To better understand the underlying phenomenon giving an advantage to the woven chips, a finite element model was developed to predict the mechanical properties obtained from a standard tensile test. DLF chips were modelled based on a voxel method where random chip positions were generated by an algorithm developed in this work. ANSYS® software was utilized to model the non-linear response associated with progressive damage of the composite. The maximum stress and the Puck failure criteria were employed to define damage initiation for the woven and UD fibres, respectively. Tensile modulus predictions for both types of chips showed good results when compared to experimental data. Strength predictions for the UD fibres also showed good correlation with experimental results, but the model overestimated the strength of the woven-fibre DLF composite. It appeared that the failure of the UD-fibre composites was associated with matrix failure (transverse tension and in-plane shear). Woven-fibre composites, however, showed damage modes linked to both fibre failure (longitudinal tension) and matrix failure (transverse tension and in-plane shear).

## Introduction

Compression moulded discontinuous-long-fibre (DLF) composites research has recently generated a lot of interest in the aerospace and automotive industries. DLF composites show great potential for recycling continuous-fibre off-cuts and remnants from manufacturing by remoulding the scraps.^1,2^ The DLF composites architecture takes advantage of the high volume fraction ( $v_{f}$ ) of the pre-impregnated continuous fibres, which ranges from 50–60%, while offering the possibility to form complex shapes,^3^ thus helping to bridge the gap between continuous-fibre and short-fibre composites.^4^ DLF composites start as a continuous-fibre prepreg tape, which is cut and slit into chips. These chips are then compression moulded into the required geometry. Studies show that DLF composites may have stiffnesses comparable to those of continuous-fibre quasi-isotropic laminates, but their strength is significantly lower.^3,5^

Early studies suggested that in-plane isotropy can be achieved in DLF composites.^3^ However, recent studies have shown that when chip flow is encountered during moulding, in-plane isotropic properties can no longer be assumed.^6–9^ Chip flow leads to fibre alignment in the direction of flow, creating a highly anisotropic material with lower properties perpendicular to the flow. Furthermore, using digital imaging correlation, some researchers have observed highly nonuniform strain fields caused by irregularity in fibre alignment.^5,10^ Weak points in the material due to unpreferred chip orientations are the source of serious concern in load-bearing components. Weak points also increase variability in mechanical properties, as shown by Feraboli et al.^6^

A recent study revealed that utilizing woven-fibre chips instead of unidirectional-fibre chips in DLF composites greatly reduces the variability in mean mechanical properties such as the tensile strength and modulus.^11^ The study also indicated that woven-fibre chips yield equal or better performances than UD-fibre chips in DLF composited subjected to tensile or bending tests. Micrographs of the failure region of the specimens suggested that the failure of the UD-fibre DLF composites was predominantly caused by high shear stresses between chips.

To better understand the underlying phenomena giving the advantage to the woven-fibre chips and to predict the mechanical behaviour of DLF composites, a finite element model (FEM) was developed in this study. Few models have been presented in the literature to simulate the behaviour of DLF composites. A model proposed by Selezneva,^12^ where 2D solid elements were employed for simplicity, showed promising results. In that FE model, the randomness of the DLF composite was included by partitioning the composite and assigning random chip orientations and stacking sequences to each partition. These stack ups were defined by an algorithm and the calculated ABD matrix from classic laminate theory were specified for each partition. Hashin’s failure criteria were used to determine damage initiation in the progressive damage response of the composite. That study was limited to the 2D response due to the selected element type.

The present study proposes using 3D elements to better represent the behaviour of DLF composites. An algorithm developed to generate the random position of the DLF chips is described hereafter. This algorithm was utilized to generate a FEM that predicted the results of standard tensile tests with DLF specimens. Model validation was performed by comparing the FEM results to experimental results from a previous study.^11^ Thereafter, the proposed model was used to compare the failure modes predicted in UD- and woven-fibre DLF composites.

## Discretization of the computational domain

The discretization of the geometry was carried out with a method inspired by the one used by Selezneva.^12^ However, the model developed here is composed of 3D solid brick elements, which makes it possible to evaluate the interlaminar stresses and the effect of the local stacking sequence of the pre-impregnated fibre chips. In this procedure, the geometry must previously be discretized with a structured mesh for easy element identification. The principle of the method is to assign an in-plane orientation to each of the elements in the model to represent the chip distribution. To accomplish this task, an algorithm was developed to position the chips randomly within the domain.

**Figure 1.**
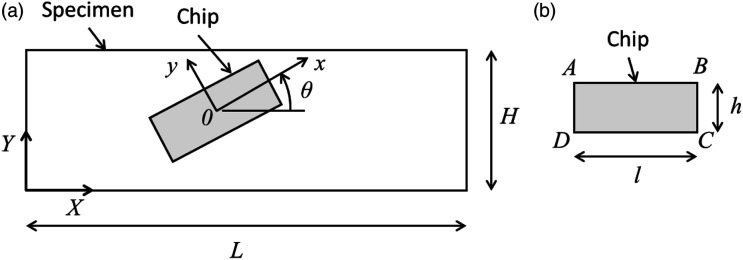
(a) Random positioning of the chips. (b) Chips dimensions.

For this model, ANSYS SOLID185 eight-node elements were chosen.^13^ The elements in-plane dimensions in such models must be significantly smaller than the chips for good accuracy, as shown by Selezneva.^12^ The size of the elements selected for this work was 0.5 mm × 0.5 mm × t (where t is the thickness of the chips), which constituted a good compromise between the accuracy of the model and the calculation time. The procedure can be viewed as one in which chips are placed arbitrarily one-by-one in the mould until it is completely filled. This is accomplished by successively assigning in-plane orientations to a group of elements representing a chip, until all elements have been processed. Each chip is first given a completely random orientation ( θ ) and position ( X , Y ) in the XY plane, as shown in Figure 1. The constructed model represents a tensile specimen for which the X position varies from 0 to L , the Y position varies between 0 and H and the chip orientation angle varies between −90° and 90°. Uniform statistical distributions were used for the position and orientation of the chips. The lengths ( L ) and width ( H ) were defined as 150 mm and 25 mm, respectively, to represent the experimental tests from a previous study.^11^ Chips dimensions were set at 12.7 mm × 12.7 mm, as in the experimental tests.

Once a chip is positioned and oriented, the elements within the area covered by that chip in the XY plane are identified (i.e., the numbers of those elements are identified). The in-plane coordinates of each of the corners (*A*, *B*, *C* and *D*) of the chip are

(1) $$X_{A}=x_{0}-\left(\frac{l}{2}\cos\theta \right)-\left(\frac{h}{2}\sin\theta \right)\;X_{C}=x_{0}+\left(\frac{l}{2}\cos\theta \right)+\left(\frac{h}{2}\sin\theta \right)$$

$$Y_{A}=y_{0}-\left(\frac{l}{2}\sin\theta \right)+\left(\frac{h}{2}\cos\theta \right)\;Y_{C}=y_{0}+\left(\frac{l}{2}\sin\theta \right)-\left(\frac{h}{2}\cos\theta \right)$$

$$X_{B}=x_{0}+\left(\frac{l}{2}\cos\theta \right)-\left(\frac{h}{2}\sin\theta \right)\;X_{D}=x_{0}-\left(\frac{l}{2}\cos\theta \right)+\left(\frac{h}{2}\sin\theta \right)$$

$$Y_{B}=y_{0}+\left(\frac{l}{2}\sin\theta \right)+\left(\frac{h}{2}\cos\theta \right)\;Y_{D}=y_{0}-\left(\frac{l}{2}\sin\theta \right)-\left(\frac{h}{2}\cos\theta \right)$$

where subscript *0* refers to the centroid of the chip being treated.

Elements in the area covered by a chip meet the criterion

(2) ABe+ADe+DCe+BCe≤l·h

where

$$ABe=\left|\frac{\left(X_{A}\cdot Y_{B}\right)+\left(X_{B}\cdot Y_{e}\right)+\left(X_{e}\cdot Y_{A}\right)-\left(Y_{A}\cdot X_{B}\right)-\left(Y_{B}\cdot X_{e}\right)-\left(Y_{e}\cdot X_{A}\right)}{2}\right|$$

$$ADe=\left|\frac{\left(X_{A}\cdot Y_{C}\right)+\left(X_{C}\cdot Y_{e}\right)+\left(X_{e}\cdot Y_{A}\right)-\left(Y_{A}\cdot X_{C}\right)-\left(Y_{C}\cdot X_{e}\right)-\left(Y_{e}\cdot X_{A}\right)}{2}\right|$$

$$DCe=\left|\frac{\left(X_{C}\cdot Y_{D}\right)+\left(X_{D}\cdot Y_{e}\right)+\left(X_{e}\cdot Y_{C}\right)-\left(Y_{C}\cdot X_{D}\right)-\left(Y_{D}\cdot X_{e}\right)-\left(Y_{e}\cdot X_{C}\right)}{2}\right|$$

$$BCe=\left|\frac{\left(X_{D}\cdot Y_{B}\right)+\left(X\cdot Y_{e}\right)+\left(X_{e}\cdot Y_{D}\right)-\left(Y_{D}\cdot X_{B}\right)-\left(Y_{B}\cdot X_{e}\right)-\left(Y_{e}\cdot X_{D}\right)}{2}\right|$$

represent the areas of the triangles formed by connecting the corners of the chip to the centroid of the evaluated element $\left(x_{e},y_{e}\right)$ . A given element is considered as part of a chip if the sum of these areas is less than or equal to the area of the chip.

**Figure 2.**
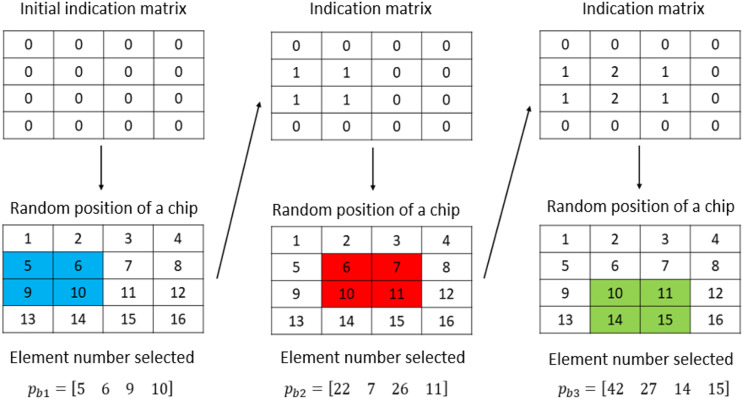
Example of a simple model having 16 elements, where three chips are positioned randomly. For simplicity, chip orientation is not considered in this illustration.

The first chip to be placed will always occupy space associated with elements from the first layer. Thereafter, when a chip (or part of a chip) is placed in an occupied space, there will be superposition. To determine the Z position occupied by an element of chip, a Z -position indicator matrix is defined. This matrix is first initialized to zero, indicating that the first chip belongs to the initial layer of elements. Each time a chip is placed, all elements in the area covered by that chip have their *Z*-position indicator increased by 1. With a structured mesh, this scheme readily links a given chip to element numbers in the model. The number of each element representing a chip is given by

(3) $$E_{num}=E_{XY}+I_{zpos}\cdot Q_{XY}$$

where $E_{XY}$ is the number of the homologous element in the first layer, $I_{zpos}$ is the *Z*-position indicator of the element of interest, and $Q_{XY}$ is the total number of elements per layer. Figure 2 shows a simple example of a model having 16 elements in which three chips are successively being randomly positioned. Chip orientation is not considered in the illustration for simplicity. This figure shows the evolution of the *Z*-position indicator matrix and how the element numbers are identified when there are chips stacked on top of each other. Initially the matrix of *Z*-position indicators consists of only zeros. In the first step, a chip is positioned and the numbers of the elements ( $p_{b1}$ ) covered by this chip are obtained from equation (3). Subsequently, the *Z*-position indicator matrix is updated. The second chip is then randomly placed and its element numbers ( $p_{b2}$ ) are found based on the newly updated *Z*-position indicator matrix. The process continues until the discretized volume is completely filled.

**Figure 3.**
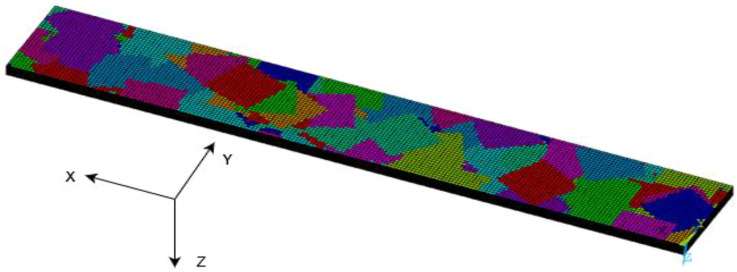
Example of a discretized model. Colours represent the coordinate systems assigned to the elements.

Once an element is linked with a chip, the randomly allocated chip angle θ is assigned to this element by defining a local coordinate system based on the angle of the chip. An example of a discretized model is shown in Figure 3. The thickness of the elements must be equal to the thickness of the chips. However, the length and width of the chips need to be specified. This makes it possible to model virtually any size of chips while keeping good accuracy, as long as the element size is significantly smaller than the chip size. In the work presented herein, the FEM mesh consisted of 22 layers of 0.14 mm thickness for the UD-fibre specimen and 12 layers of 0.26 mm thickness for the woven-fibre specimen. These values correspond to the actual specimens that were experimentally tested.^11^

In the FE model, since adjacent elements share nodes, mating chips are considered perfectly bonded to each other, surface-to-surface or edge-to-edge, depending on the case. Therefore, load is transferred between chips by surface-to-surface shear but also by edge-to-edge tension. This reduces the stress concentration that would normally appear in a shear-lag zone. However, in the physical specimens, the chips get thinner near their edges,^11^ where they are “squeezed” between their upper and lower neighbours, and the stress concentration is effectively reduced. The simplifications made in the FE model, while ensuring computational efficiency and modelling simplicity, do not affect its capacity to predict the behaviour of the DLF composites under study.

## Model definition

### Mechanical behaviour

Two types of fibre-reinforced thermoplastics (Toray Cetex® TC1000 PEI-matrix) were considered for this analysis: a UD-fibre DLF composite and a woven-fibre DLF composite. The reinforcements were AS4 (UD) and FT300B (woven) carbon fibres. The 5 Harness Satin weave architecture had 20 fill and 20 warp counts per inch. The mechanical properties of the composite chips are presented in Table 1. Some of the properties of the materials were estimated based on similar materials (see asterisks in Table 1). Properties that are not asterisked were taken from the material supplier’s data.^14^

**Table 1.** Mechanical properties of the composite chips used for numerical analysis.^14^

	UD	Woven
$E_{11}$ [GPa]	120^*^	56^*^
$E_{22}$ ( $E_{33}$ ) [GPa]	9^*^	57^*^
$G_{12}$ ( $G_{13}$ ) [GPa]	5.2	4.5^**^
$G_{23}$ [GPa]	4	4^**^
$\nu_{12}$ ( $\nu_{13}$ )	0.29	0.05^**^
$\nu_{23}$	0.4	0.3^**^
$X_{t}$ [MPa]	2241	682^*^
$X_{c}$ [MPa]	1200	514^**^
$Y_{t}$ [MPa]	92	740^*^
$Y_{c}$ [MPa]	92	705^**^
$S_{12}$ [MPa]	141	136^**^

^*^Measured property.

^**^Estimated property.

A progressive damage model, already available in ANSYS, was included to represent the non-linear fracture behaviour of the composite. Three variables track damage and reduce the rigidity of the elements that have experienced failure: damage in direction 1 ( $d_{1}$ ), damage in direction 2 ( $d_{2}$ ) and in-plane shear damage ( $d_{12}$ ). The resulting stress-strain relationship is given by

(4) σ=[D]ε

where the elasticity matrix is

$$\left[D\right]={\left[\begin{array}{cccccc} \frac{C_{11}}{\left(1-d_{1}\right)} & C_{12} & C_{13} & 0 & 0 & 0\\ C_{21} & \frac{C_{22}}{\left(1-d_{2}\right)} & C_{23} & 0 & 0 & 0\\ C_{31} & C_{32} & \frac{C_{33}}{\left(1-d_{2}\right)} & 0 & 0 & 0\\ 0 & 0 & 0 & \frac{C_{44}}{\left(1-d_{12}\right)} & 0 & 0\\ 0 & 0 & 0 & 0 & \frac{C_{55}}{\left(1-d_{12}\right)} & 0\\ 0 & 0 & 0 & 0 & 0 & \frac{C_{66}}{\left(1-d_{12}\right)} \end{array}\right]}^{-1}$$

### Puck’s model

To determine whether the UD-fibre composite material has suffered damage, the modified Puck failure criteria^13^ were used. According to this model, the failure criterion in direction 1 (i.e., direction parallel to the fibres) is defined by

(5) $$f_{1}=\left|\frac{\sigma_{1}}{X}\right|$$

where $\sigma_{1}$ represents the stress in direction 1 and

$$X=\left\{ \begin{array}{c} X_{c},\sigma_{1}<0\\ X_{t},\sigma_{1}\geq 0 \end{array}\right.$$

is the compressive or tensile strength in direction 1.

The failure criterion in direction 2 (i.e., failure of the matrix) is defined by

(6) $$f_{2}=\frac{\sigma_{2}^{2}}{Y_{t}Y_{c}}+\frac{\tau_{12}}{S^{2}}+\left(\frac{1}{Y_{t}}+\frac{1}{Y_{c}}\right)\sigma_{2}$$

where $\sigma_{2}$ and $\tau_{12}$ represent the stress in direction 2 and the shear stress in plane 12, respectively. $Y_{t}$ and $Y_{c}$ are the tensile and compressive strengths in direction 2, respectively, and S is the shear strength in plane 12. Puck’s model is therefore defined as

$$f=\max\left(f_{1},f_{2}\right)$$

The element fails when f is greater than 1.

### Maximum stress model

To determine whether the woven-fibre composite material has suffered damage, the maximum stress model^13^ was used. This model was used for woven fibres because Puck’s failure criteria considers that there are no fibres in direction 2, which is not the case for woven fibres. The failure criteria in plane 12 are defined by

(7) $$f_{1}=\left|\frac{\sigma_{1}}{X}\right|\;f_{2}=\left|\frac{\sigma_{2}}{Y}\right|\;f_{12}=\left|\frac{\tau_{12}}{S}\right|$$

where

(8) $$X=\left\{ \begin{array}{c} X_{c},\;\sigma_{1}<0\\ X_{t},\;\sigma_{1}\geq 0 \end{array}\right.\;\;\;Y=\left\{ \begin{array}{c} Y_{c},\;\sigma_{2}<0\\ Y_{t},\;\sigma_{2}\geq 0 \end{array}\right.$$

The maximum stress model is therefore defined as

$$f=\max\left(f_{1},f_{2},f_{12}\right)$$

The element fails when f is greater than 1.

These damage criteria do not consider complex failure mechanisms such as tow-tow delamination or transverse cracking within tows that may occur in woven-fabric composites.^15^ This homogeneous (effective continuum) properties modeling approach was selected for its ease of implementation, its efficiency, and its relatively good accuracy, which are important factors for industrial applications.

### Damage variables

Damage variables are used to reduce the stiffness of the material at the locations where failure has occurred. This reduction in stiffness represents progressive damage, i.e., the evolution of damage in the material. In the present model, damage variables can take one of two possible values, 0 or 0.95, where 0 represents no damage and 0.95 represents the maximum damage to the element. This is mathematically expressed as

(9) $$d_{1}=\left\{ \begin{array}{c} 0,\;f_{1}<1\\ 0.95,\;f_{1}\geq 1 \end{array}\right.\;\;d_{2}=\left\{ \begin{array}{c} 0,\;f_{2}<1\\ 0.95,\;f_{2}\geq 1 \end{array}\right.\;\;d_{12}=\left\{ \begin{array}{c} 0,\;f_{12}<1\\ 0.95,\;f_{12}\geq 1 \end{array}\right.$$

### Conditions of the analysis

**Figure 4.**
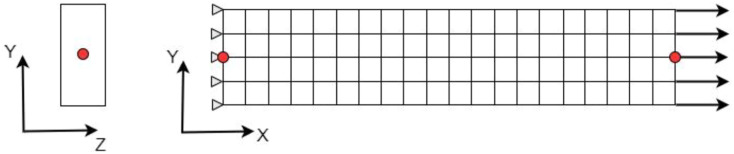
Prescribed boundary conditions of the finite element model.

Due to the random chip distribution in the model, several simulations were performed for each chip type, UD and woven. This allowed to perform statistical analyses on the results, as well as to validate the model by comparing its predictions with previous experimental results.^11^ The boundary conditions shown in Figure 4 were prescribed to represent the conditions of the tensile tests. Uniform displacement in positive X -direction were applied to the nodes at the right end, while the nodes at the left end were fixed in the X -direction. To prevent rigid body motion in the Y and Z directions, single nodes at each end of the model were fixed. These nodes are represented by the red dots in Figure 4. These boundary conditions were imposed to represent a sample fixed in the jaws of a tensile testing apparatus.

For the simulation, automatic step adaptation was enabled, with an initial step of 0.01, a minimum step of 10^−4^ and a maximum step of 0.1. Convergence difficulties were mitigated by a viscous regulation scheme with a stabilization factor of 10^−4^, which ensured that the tangent stiffness matrix was positively defined for sufficiently small increments. Complete material failure was assumed when the model diverged.

## Results and discussion

**Figure 5.**
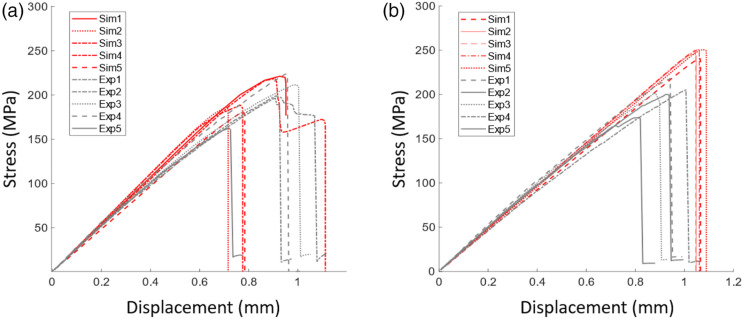
Tensile curves of experimental and simulated specimens for (a) unidirectional chips and (b) woven chips. The vertical axis represents the effective stress on the tensile specimen. The red curves, identified as Sim1…5 in the legend, are results from the simulations. The gray curves (Exp1…5) represent experimental results.

**Figure 6.**
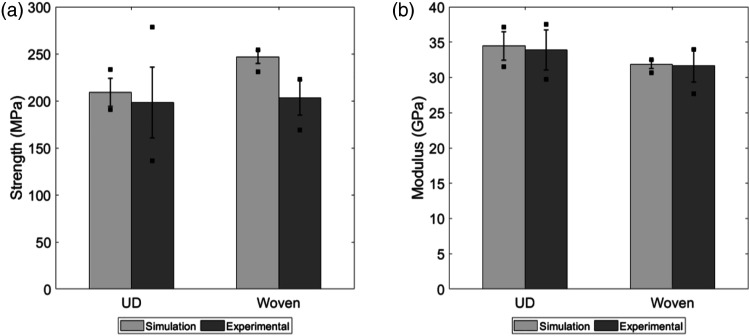
Comparison of the mechanical properties of unidirectional and woven chips. (a) Tensile strength and (b) tensile modulus.

Experimental and predicted tensile curves for the two materials are shown in Figure 5. In order not to overload the graphs, only five curves of each type are presented. To better compare the results, mechanical properties obtained from these curves are shown in Figure 6, where the bars represent the average, the error bars represent the standard deviation, and the square points represent the extreme values. Tensile strengths and moduli are compared in these graphs. The data of Figure 6 is based on the results of ten simulations for the UD chips and ten simulations for the woven chips.

An analysis of variance (ANOVA) is used to validate the data, with an alpha limit of 5%. The ANOVA analysis compares numerical results with experimental values. The *p*-values of these analyses are presented in Table 2.

**Table 2.** P-values obtained for the comparison between numerical and experimental analyses.

	*p*-value
Strength UD fibres	0.41
Strength woven fibres	<0.05
Modulus UD fibres	0.61
Modulus woven fibres	0.79

The results of Figure 6 show that the model can accurately predict the modulus of composites made with both types of chips (Figure 6(b)). However, the model significantly overestimates the strength in the case of the woven fibres (Figure 6(a)). Strength predictions strongly depend on the damage model and this result may be attributable to the fact that the maximum stress criteria were employed for woven chips, as discussed in Section *Maximum stress model*. On the other hand, Puck’s model adequately represents the behaviour of the UD-chip composite. Despite the overestimation of the tensile strength of the woven-chip composite, it is still possible to analyze the failure modes of both types of chips by observing trends. The variability in the test results is reasonably well predicted by the model, except in the case of the strength of the UD-chip composite, where the variability from experimental testing is large.

The numerical model predicts a trend similar to the one observed from the experimental tests of Belliveau et al.^11^ Woven-fibre chips, having “more isotropic” in-plane mechanical properties, significantly reduce the variability of the strength and modulus of the DLF composite compared to highly anisotropic UD-fibre chips.

**Figure 7.**
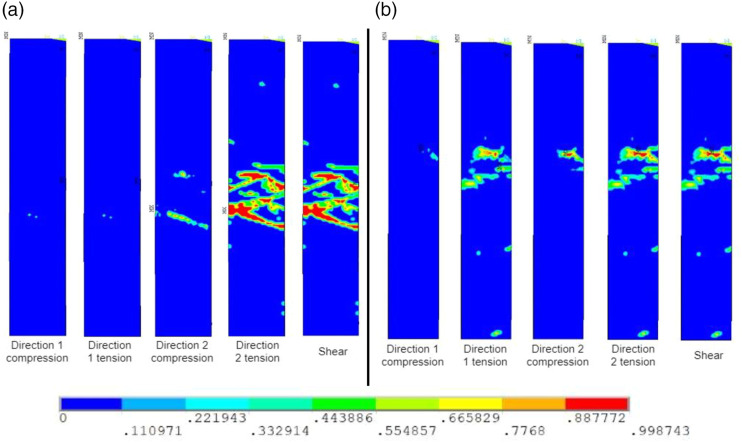
Failure modes predictions for the (a) UD-fibre DLF composite and (b) woven-fibre DLF composite. The scale represents the damage, where 0 means no damage and 1 means complete rupture of the element.

Figure 7 shows predictions of failure from the five possible modes: tension and compression in directions 1 and 2 as well as in-plane shear 12. Figure 7 reveals that the failure of specimens with UD chips is mainly caused by the rupture in direction 2 of the chips, i.e., the direction perpendicular to the fibres. Shear failure is also very apparent. However, fibre breakage is practically non-existent. On the other hand, specimens with woven chips exhibit significant damage in both directions (1 and 2), as well as damage caused by shear. Remembering that there are fibres aligned with both directions in woven chips, these chips suffer much more uniform damage compared to UD-fibre chips, thus reducing the potential for weaknesses in the specimens.

**Figure 8.**
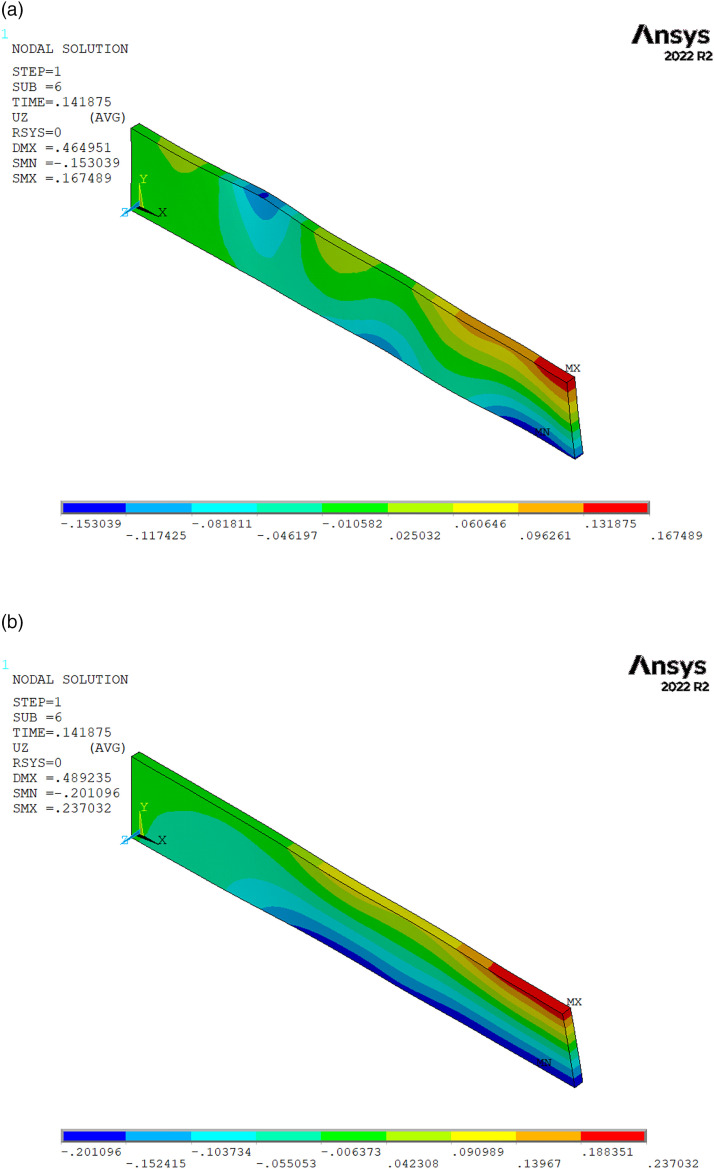
Out-of-plane displacements (in mm) for an axial elongation of 0.43 mm. (a) UD-fibre chips (b) woven-fibre chips.

By using 3D elements, the full 3D behaviour of the specimen can be captured, with out-of-plane displacements, which are shown in Figure 8. The displacements shown correspond to an axial elongation of 0.43 mm of the specimen, for which no damage has yet occurred. The UD-fibre specimen shows some positive and negative out-of-plane displacements resulting in surface waviness. This is attributable to the significant stiffness variations within the specimen. By contrast, the woven-fibre specimen yields a much smother out-of-plane displacement distribution along its axis. The twist in the samples is attributable to the non-symmetric, random layup.

## Conclusion

This study presented a simple but efficient way to simulate the mechanical behaviour of DLF composites made from pre-impregnated chips. The proposed FEM, based on 3D solid elements, was fully developed using ANSYS software without requiring external means to discretize the domain. By using progressive damage features, the model can predict the strength and modulus of DLF specimens. The developed model is simple to implement and require relatively low calculation time.

The proposed model was used in a comparative analysis to understand the difference in behaviour between DLF composites fabricated from UD chips and woven chips. From this analysis, it was concluded that the improved isotropy of the in-plane properties provided by the woven-fibre chips have the potential to reduce variability and therefore increase the mechanical performance of DLF composites. The failure modes observed were mainly matrix (chip direction 2) and shear failure for the UD-fibre DLF composite compared to damage in chip directions 1 and 2 for the woven-fibre DLF composite.

## References

[1] Pickering SJ . Recycling technologies for thermoset composite materials - current status. Compos Part A 2006; 37: 1206–1215.

[2] Leblanc D Landry B Jancik M , et al. Recyclability of randomly-oriented strand thermoplastic composites. 20th International Conference on Composite Materials. Copenhagen, July 2015.

[3] Feraboli P Peitso E Deleo F , et al. Characterization of prepreg-based discontinuous carbon fiber/epoxy systems. J Reinf Plast Compos 2009; 28: 1191–1214.

[4] Eguémann N . Processing of characterisation of carbon fibre reinforced PEEK with discontinuous architecture. 16th European Conference on Composite Materials ECCM16. Seville, June 2014.

[5] Feraboli P Peitso E Cleveland T , et al. Modulus measurement for prepreg-based discontinuous carbon fiber/epoxy systems. J Compos Mater 2009; 43: 1947–1965.

[6] Feraboli P Cleveland T Ciccu M , et al. Defect and damage analysis of advanced discontinuous carbon/epoxy composite materials. Compos Part A Appl Sci Manuf 2010; 41: 888–901.

[7] Belliveau R Léger É Landry B , et al. Measuring fibre orientation and predicting elastic properties of discontinuous long fibre thermoplastic composites. J Compos Mater 2020; 55(3): 321–330.

[8] Léger É Landry B LaPlante G . High flow compression molding for recycling discontinuous long fiber thermoplastic composites. J Compos Mater 2020; 54: 3343–3350.

[9] Sommer DE Kravchenko SG Denos BR , et al. Integrative analysis for prediction of process-induced, orientation-dependent tensile properties in a stochastic prepreg platelet molded composite. Compos Part A Appl Sci Manuf 2020; 130: 105759.

[10] Selezneva M Lessard L . Characterization of mechanical properties of randomly oriented strand thermoplastic composites. J Compos Mater 2016; 50: 2833–2851.

[11] Belliveau R Landry B LaPlante G . Comparative study of the mechanical properties of woven and unidirectional fibres in discontinuous long-fibre composites. J Therm Compos Mater 2022; doi:10.1177/08927057221091084. PMC10235529 37275339

[12] Selezneva M. Experimental and theoretical investigations of mechanical properties of Randomly-Oriented Strand (ROS) composites*.* Doctoral Thesis, McGill University, Canada, 2015.

[13] Ansys Help [Online]. Available: www.ansyshelp.ansys.com (accessed 8 September 2021).

[14] Toray Cetex® TC1000 product data sheet. Morgan Hill, California: Toray Advanced Composites, 2019.

[15] Meyer C O’Brien DJ Haque BZ , et al. Mesoscale modeling of ballistic impact experiments on a single layer of plain weave composite. Compos Part B Eng 2022: 235.

